# Care of Children’s Teeth

**Published:** 1910-10-15

**Authors:** Thos. E. Weeks


### CARE OF CHILDREN'S TEETH.

BY THOS. E. WEEKS, D.D.S.

First, it is absolutely necessary that we gain the confidence of the child. This is not always an easy task, especially if the parents have threatened the child with a visit to the dentist as a punishment for some offense, which is sometimes thoughtlessly done. Adults are almost always to blame when the child regards the dentist with fear; so many seem to delight in magnifying their suffering from dental operations. When this is done in the presence of children it seldom fails to impress them unfavorably. I remember one middle-aged gentleman, an attorney, who invariably slept through the major portion of every operation, but who always entertained any acquaintance who might be in the waiting-room when he left the chair with a graphic account of his sufferings. On several occasions there were children present; then he would direct his story and his sympathy to them. Children seldom have fear of us unless it has been instilled into them by their elders, and sympathy usually increases our difficulties. For this reason I try to have them come alone, and under no condition will I permit the mother or father to stand beside the chair.

Most children are willing to be shown. and a little patience and tact combined with honesty will usually succeed. If you can impress the child at the first sitting that you have accomplished something without inflicting pain, you have gone far in gaining his or her confidence.

*Never deceive a child* If you promise them not to hurt, keep your promise even if you fail to accomplish all you desired. If some pain is unavoidable, wait until some degree of confidence is established, then tell them so, and ask their co-operation, promising to desist at their request. If they *know* that you will keep your promise and obey such requests, they will feel that *they* are masters of the situation, and their pride will induce them to bear such discomfort as they know is unavoidable.

The experience of most of us has been with children coming from families having some appreciation of the importance of sound teeth, and it is quite a different proposition when we come to deal with children who come from homes where the only idea of a dentist is that he "pulls teeth," "hurts dreadfully," and is a thing to be avoided. This makes it all the more necessary that we avoid inflicting pain in the beginning.

### METHODS AND MATERIALS FOR PRESERVING CHILDREN'S TEETH.

Let us look into the matter closely and see how nearly we agree on methods and materials used in conserving the teeth of children. If we make freedom from pain the object of paramount importance in our service to children, as I believe we should, can we satisfy our ideals as to the character of the operation?

Gold has been our standard of excellence in filling materials, and those high in authority have told us that children's teeth are as capable of sustaining and retaining a metallic filling as those of adults. Many of us have filled the teeth of very young children with gold, or when the parents were unable to afford gold, we have used amalgam, to satisfy our conception of excellence and permanence. The indestructibility and integrity of the material employed in filling a tooth is not the only factor that determines the permanence of the operation. Time will not permit a full consideration of this subject, but we may mention some of the causes of failure when gold or amalgam is used in children's teeth.

### CAUSES OF FAILURE OF GOLD AND AMALGAM FILLINGS IN CHILDREN'S TEETH.

First, let us consider the condition in case of failures, aside from those cases where the filling is lost in bulk owing to insufficient anchorage. Without considering the cause or reason for recurrent caries around or beneath a filling, whether it be due to faulty preparation, failure to sterilize infected dentin, faulty insertion of the material, or failure to extend the cavity margins into immune areas, the condition is practically the same, *i. e.*, the filling is *in situ*, with more or less destruction of enamel and dentin around or beneath the filling. The very presence of the filling affords protection to the destructive organisms.

We do not deny that a gold or amalgam filling *can* be made even in the teeth of very young children, so that it will remain and fulfill its office permanently, but is it always wise to do it?

### INDICATIONS FOR PERMANENT OR TEMPORARY FILLINGS.

Much has been written on the subject of "humane dentistry," and if we are to establish and maintain a good mental attitude on the part of children toward us we *must* be humane. Let us remember that our whole duty to children has not been discharged when we have filled the cavity perfectly. Let us get away from the idea that we must in every case attempt a so-called "permanent" filling There are some conditions which contra-indicate it; let us not ignore them. Because of conditions beyond our control, materials considered as temporary often make the most permanent operations in children's teeth.

### AN INADVISABLE METHOD OF FILLING.

Take a not unusual case. A child of eight presents with the first permanent lower molar showing a mesioocclusal cavity so far progressed that when it is opened and the *softened* dentin is removed, the pulp is covered with only a thin layer of dentin, which is undoubtedly infected. There are still those practitioners who, to satisfy their ideas, flood the cavity with creosote, phenol, or some similar drug, fill the deeper portion of the cavity with zinc oxyphosphate, and complete the filling with amalgam; all this at one sitting, or at best after sealing in some anodyne dressing for a day or two. In order to do this thoroughly the application of the rubber dam is required, as is also more or less cutting of dentin and enamel to provide proper anchorage for the filling and to place the margins of the cavity in safe areas, and further, the final finishing and polishing of the amalgam. The average operator would require from forty-five minutes to an hour to do this as it should be done. To consider anything short of the most thorough technique would be an insult to this body.

If the operator who proceeds in the way described above would stop to consider the minute anatomy and pathology of the tooth, and the possible changes that may take place in the pulp under such treatment, he would seek, first, a substance for the filling of the deeper portion of the cavity which would be sedative to the pulp, render the infected dentin aseptic, and protect the pulp from thermal changes. Do phenol and zinc oxyphosphate fulfill these requisites.? Second, he would seek a material for the remainder of the filling which could be inserted easily, and which would protect the walls of the cavity from recurrent caries, even though it may need occasional restoration of contour in order to maintain proper contact with the adjoining tooth. Both he and the little patient would be less fatigued if such a procedure could be performed in half the time, beside avoiding the use of the rubber dam.

### SUGGESTIONS FOR A RAPID FILLING METHOD.

Let us now present another picture of the same case, giving the outlines of the technique, so that it may stand out more clearly.

The unsupported enamel is cut away with sharp chisels. The softened dentin is removed with a pair of right and left spoon excavators, that must be very sharp and as large as the cavity will permit. The cavity is then ready for the filling without further shaping. Mix some iodoformogen cement rather stiff, and set it aside until all other preparations are complete. Then mix some copper oxyphosphate according to directions, Ames' Original, if the patient has experienced any discomfort, otherwise the New Process may be used. Wash the cavity with a warm sodium carbonate solution, apply cotton rolls to exclude the moisture, wipe the cavity dry with bibulous paper or absorbent cotton pledgets, fill the deeper portions, covering all or nearly all of the dentinal walls with the iodoformogen, being careful to keep it off the enamel walls. This will set promptly when inserted in the cavity. (If there is danger from thermal changes, a disk of asbestos felt may be placed over the iodoformogen while it is still plastic.) The copper oxyphosphate is then introduced with platinoid or German silver instruments. It may be hardened with a blast of warm air, or by covering it with a piece of hot base-plate gutta-percha, which has been previously provided. As soon as the cement becomes sufficiently hard to resist solution by the saliva, the filling may be trimmed with sharp knife trimmers. This whole operation will consume little more time than it has taken to describe it.

### IODOFORMOGEN AND OXYPHOSTHATE OF COPPER FOR FILLING THE TEETH OF CHILDREN

These materials have a decidedly sedative and stimulant action upon the pulp, besides possessing antiseptic properties. Copper phosphate will restore the contour and the contact, insuring comfort in the mastication of food; it will outlast any other cement, and it will adhere so closely to the walls of the cavity as to require little or no retentive form in the cavity; because of this close adhesion it will preclude recurrent caries. Furthermore, if the contour is lost by attrition, it is easily and quickly restored by scraping the surface and adding some freshly mixed copper oxyphosphate, which will adhere to the old filling. All this can be accomplished without pain, without the use of the dreaded engine or the rubber dam, and is in thorough accord with the principles enunciated. About the only objection is that the filling becomes ebony black. This, however, should not over-balance its good qualities. Sentiment ought not to influence us in so important a matter as this. The physician does not hesitate to use strychnin and other poisons in proper doses; their toxic properties do not deter him from employing them when they are superior to other remedies, and it would seem that we, too, are justified in selecting our weapons with which to fight disease, for the same reasons.

Of course we must confine the use of copper oxyphosphate to those locations which are inconspicuous. It should not be used in pulpless teeth, for it will stain them, but it will not discolor a vital tooth.—*Cosmos.*

